# A Complicated Triad of Diabetic Ketoacidosis, Hypertriglyceridemia, and Acute Pancreatitis in a Patient With Alcohol Abuse and Undiagnosed Diabetes Mellitus

**DOI:** 10.7759/cureus.75418

**Published:** 2024-12-09

**Authors:** Kenneth J Adams, Subekshya Khadka, Diki Sherpa, Natalie Saliba

**Affiliations:** 1 Medical School, Edward Via College of Osteopathic Medicine, Auburn, USA; 2 Internal Medicine, Crestwood Medical Center, Huntsville, USA

**Keywords:** alcohol use disorder, dka, hypertriglyceridemia-induced pancreatitis, triad, undiagnosed type 2 diabetes mellitus

## Abstract

Diabetic ketoacidosis (DKA), hypertriglyceridemia, and acute pancreatitis are a rare and potentially fatal triad. This article presents a fatal case of acute pancreatitis, DKA, and hypertriglyceridemia in a patient with undiagnosed diabetes mellitus struggling with alcoholism. The patient was unresponsive to standard pancreatitis and DKA treatment protocol and progressed to develop multi-organ failure. Despite best efforts, the patient expired on day five of admission.

## Introduction

Although diabetic ketoacidosis (DKA) is common, the triad of acute pancreatitis, DKA, and hypertriglyceridemia is rare and occurs in just 4% of DKA cases [1,2]. Nair and Pitchumoni first described this phenomenon as the “enigmatic triangle” in 1997 [3]. It is a very rare first presentation of diabetes mellitus in adult patients and has been documented in younger adults [1,2,4]. This particular triad is associated with worse outcomes compared to acute pancreatitis alone [5]. The symptoms of DKA and pancreatitis overlap, which presents another obstacle to the clinician in making the appropriate diagnosis and treatment in the setting of the triad. This article presents a 29-year-old male patient with the triad who was also struggling with alcohol abuse.

## Case presentation

A 29-year-old male patient with no previous medical history presented to the emergency department with a complaint of persistent, diffuse, and dull epigastric abdominal pain that started approximately 10 hours earlier. He stated his pain worsened after eating greasy food earlier in the day. The patient vomited once shortly after being seen in the emergency department. The vomit was nonbloody and nonbilious. He denied any history of fever, chills, hematemesis, melena, hematochezia, diarrhea, constipation, dysuria, polydipsia, polyuria, or hematuria. His social history was significant for drinking a liter of vodka every day along with frequently smoking and using tobacco products.

In the emergency room, the patient’s vitals displayed no abnormalities except for an elevated blood pressure of 156/110 mmHg (Table 1). Physical examination revealed an alert and obese male patient in moderate pain. The examination displayed a mildly distended abdomen and right and left upper quadrant tenderness upon palpation. Bowel sounds were normal in all four quadrants, and there was no guarding or rebound tenderness noted. The rest of the physical exam showed no abnormalities.

**Table 1:** Five-Day Vital Signs T: temperature; HR: heart rate; BPS: systolic blood pressure; BPD: diastolic blood pressure; RR: respiratory rate

Value	Day 1	Day 2	Day 3	Day 4	Day 5	Reference range
T	97.8	98.8	102.6	94.4	96.5	97-99°F
HR	88	130	137	113	95	60-100 bpm
BPS	156	137	113	49	70	≈120 mmHg
BPD	110	77	90	32	43	≈80 mmHg
RR	18	16	30	30	30	12-20 bpm

**Figure 1:**
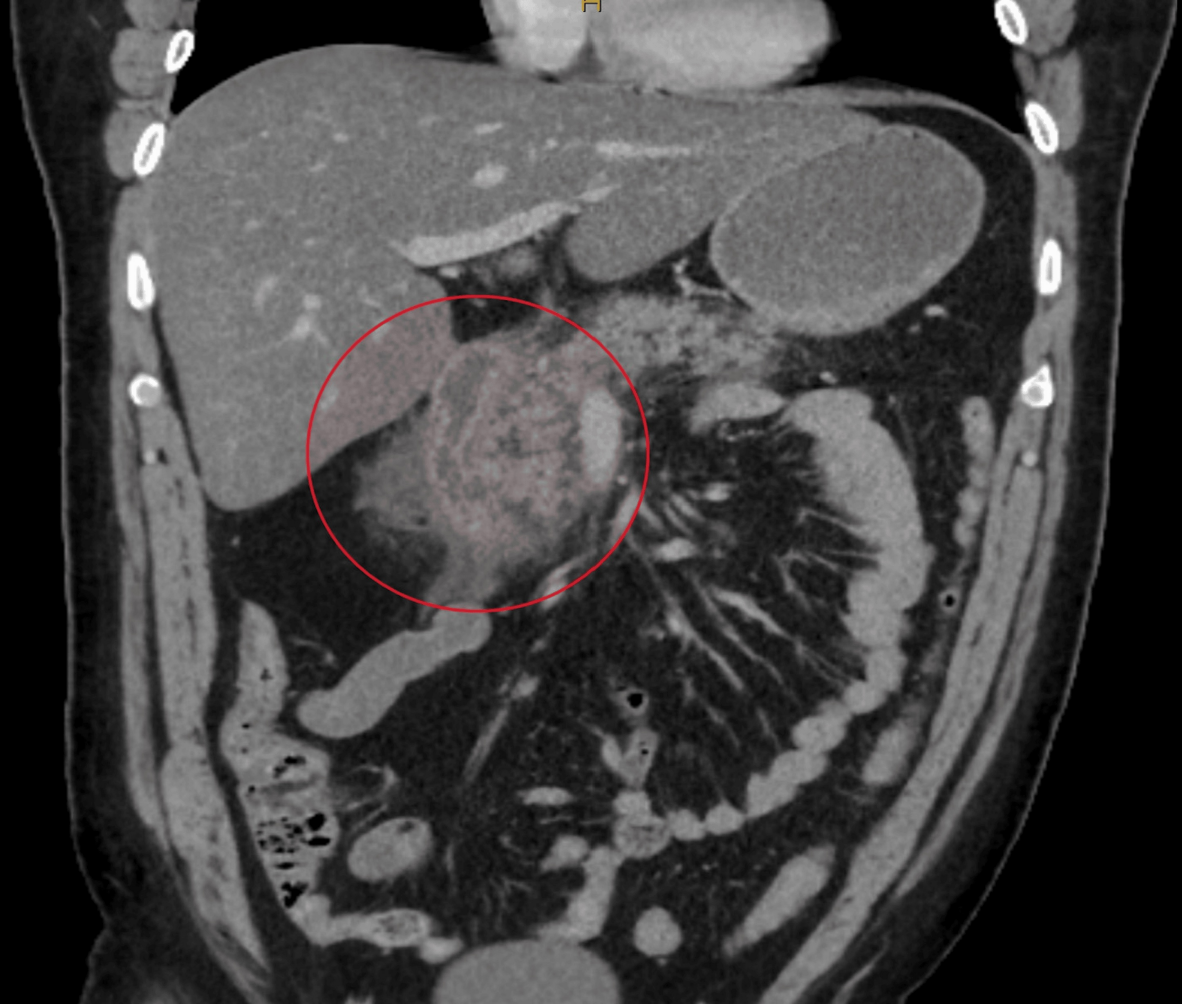
Coronal CT of the Abdomen and Pelvis Without Contrast Showing Peripancreatic Stranding, Contiguous Inflammation About the Descending Duodenum, Fluid in the Mesentery, and Hepatic Steatosis

Chemistry values on admission revealed an elevated anion gap of 19.7, lipase of 3,016 U/L, and a blood glucose level of 377 mg/dL (Table 2). Hematologic values on admission showed a white blood cell count of 13.1 Thou/μL, hemoglobin of 16.2 g/dL, hematocrit of 44.2%, and a platelet count of 350 Thou/μL (Table 3). Urinalysis revealed ketones in the urine. A CT of the abdomen and pelvis without contrast showed peripancreatic stranding, contiguous inflammation about the descending portion of the duodenum, and fluid in the mesentery suggesting acute pancreatitis (Figure 1).

**Table 2:** Significant Chemistry Values CREA: creatinine; BUN: blood urea nitrogen; AST: aspartate aminotransferase; ALT: alanine aminotransferase; ALP: alkaline phosphatase; TRIG: triglycerides; TNIH: troponin I; GLU: glucose

Value	Day 1	Day 2	Day 3	Day 4	Day 5	Reference range
Na^+^	132	125	142	143	140	136-145 mmol/L
K^+^	4.7	5.2	3.6	4.3	5.6	3.5-5.1 mmol/L
Ca^+^	6.3	6.5	5.7	5.6	<5	8.4-10.2 mg/dL
Anion gap	19.7	22.3	23.6	29.3	36.6	6.0-14.3
CREA	1	1.3	6.3	7.1	7.6	0.6-1.3 mg/dL
BUN	9	22	40	41	38	7-18 mg/dL
AST	43	-	62	14,972	632	15-37 U/L
ALT	-	100	41	5,312	6,608	12-78 U/L
ALP	132	121	78	187	219	41-137 U/L
TRIG	-	4,910	2,346	864	-	<150 mg/dL
TNIH	5	3	2,904	2,193	-	0-79 ng/L
GLU	377	381	204	222	414	70-100 mg/dL
Lipase	3,016	-	-	-	-	13-75 U/L

**Table 3:** Significant Hematologic Values WBC: white blood cell; HGB: hemoglobin; HCT: hematocrit; PLT: platelet

Value	Day 1	Day 2	Day 3	Day 4	Day 5	Reference range
WBC	13.1	11.9	9.5	13.3	10.7	4.0-10.6 Thou/μL
HGB	16.2	18	18.9	13.5	8.8	12.2-17.5 g/dL
HCT	44.2	53.2	57	43.3	28.4	35.6%-50.6%
PLT	350	342	369	15	19	145-418 Thou/μL

After a thorough workup, a diagnosis of acute pancreatitis and DKA was made based on the patient's history, examination, laboratory values, and imaging results. The patient was started on an aggressive fluid resuscitation program along with managing his pain and nausea with analgesics and antiemetics. Also, prophylactic antibiotics were administered to prevent secondary infection. The patient was moved to the ICU and was started on an insulin drip. Plasmapheresis was discussed but could not be performed at the facility due to its unavailability. A few hours after admission, the patient’s HbA1c and triglyceride levels returned elevated at 11.9% and 4,910 mg/dL (Table 2). Arterial blood gas was ordered after moving him to the ICU, and it revealed an elevated anion gap metabolic acidosis with a pH of 7.22 (Table 4). Despite initial management, the patient’s condition quickly deteriorated requiring intubation on day two due to worsening respiratory status. He went on to develop multiple complications over the next few days including (1) distributive shock, (2) kidney failure, (3) liver failure, (4) infection, and (5) myocardial infarction (Tables 1-4). Dialysis was attempted but could not be performed due to the patient’s unstable condition. He was started on multiple pressors for his distributive shock and eventually placed on the maximum amount. An MRI was planned on day three for possible brain death but could not be performed due to the patient's unstable condition. Despite all efforts, the patient expired on day five of admission.

**Table 4:** Significant Arterial Blood Gas Values

Value	Day 1	Day 2	Day 3	Day 4	Day 5	Reference range
pH	-	7.22	7.09	6.97	6.79	7.35-7.45
PO_2_	-	83	56	<55	<55	80-105 mmHg
PCO_2_	-	30.6	51.5	60.1	55	35-45 mmHg
HCO_3_^-^	-	12.7	14.2	13.8	8.3	22-26 mmol/L
O_2_SAT%		94	74	55	45	95%-98%

## Discussion

As stated earlier, the condition known as the enigmatic triangle is rare and associated with worse outcomes [1-3,5]. It can be a fatal condition as noted in our case and other cases [6]. The condition is thought to stem from hypertriglyceridemia-induced pancreatitis caused by an insulin-deficient state in DKA [1]. Also stated in the previous article referenced is that this triad can be a rare first presentation of type 2 diabetes mellitus.

Diagnosis of this triad can be complicated due to the overlap of clinical symptoms between DKA and acute pancreatitis [7]. Acute pancreatitis is diagnosed by the revised Atlanta classification system [8]. According to the Atlanta classification system, the diagnosis of acute pancreatitis requires two out of the following three criteria: (1) abdominal pain characteristic of acute pancreatitis, (2) serum lipase greater than three times the upper limit of normal, and (3) characteristic findings of acute pancreatitis on abdominal CT, MRI, or transabdominal ultrasonography. DKA is diagnosed by an elevated anion gap metabolic acidosis, ketonuria or ketonemia, and an elevated blood glucose of greater than 250 mg/dL [9]. Many different laboratory tests are initially ordered to rule out differential diagnosis. The most important ones to include are a hepatic panel to assess for gallstone-induced pancreatitis and a lipid panel to assess for hypertriglyceridemia-induced pancreatitis. Both of these values were ordered in our patient’s case. These laboratory tests are important because they determine different routes of management.

Treatment of the triad is focused on treating both DKA and hypertriglyceridemia-induced pancreatitis. Treatment mainly consists of massive fluid resuscitation, insulin to decrease ketone production, and pain management. It has been suggested in previous literature that plasmapheresis may be an effective addition to the treatment of the triad [1,10]. Of note, treatment of DKA in itself can benefit hypertriglyceridemia-induced pancreatitis with the infusion of insulin [11]. That article discusses how insulin can help induce lipoprotein lipase (LPL) and accelerate chylomicron destruction in helping lower triglyceride levels. In our case, the patient underwent standard DKA and pancreatitis protocol. Plasmapheresis was considered but was unable to be performed at the hospital where the patient was located due to the lack of availability. It is unclear if plasmapheresis would have been of benefit in this patient’s case.

The most common causes of acute pancreatitis are gallstones occurring at a frequency of 40%, alcohol occurring at 30%, and hypertriglyceridemia occurring at 2%-5% [12]. According to this article, hypertriglyceridemia is a rare cause of acute pancreatitis relative to alcohol and gallstones. The obstacle we faced in our case was determining the initiating cause of his pancreatitis. It is difficult to distinguish if the cause was due to hypertriglyceridemia due to DKA or if it was due to his alcohol consumption. Another factor to consider is if the patient had an inherited dyslipidemia. Specifically, type Ⅰ, type Ⅳ, or type Ⅴ hereditary dyslipidemia has been known to cause hypertriglyceridemia-induced pancreatitis [13].

## Conclusions

Hypertriglyceridemia-induced pancreatitis associated with DKA is a rare event and is fatal. It is associated with worse outcomes compared to acute pancreatitis alone. Alcoholism may further exacerbate the condition as well as hereditary dyslipidemias. The addition of plasmapheresis along with standard treatment may improve patient outcomes, but more research is needed to assess the effectiveness in this particular population. The clinician should always be mindful of the possible co-occurrence of DKA and pancreatitis in an undiagnosed diabetic.
